# Combined Treatment of Non-Small-Cell Lung Cancer Using Shenyi Capsule and Platinum-Based Chemotherapy: A Meta-Analysis and Systematic Review

**DOI:** 10.1155/2020/3957193

**Published:** 2020-10-13

**Authors:** Yong Xu, Wenpan Peng, Di Han, Zhichao Wang, Cheng Gu, Fanchao Feng, Xianmei Zhou, Qi Wu

**Affiliations:** ^1^Affiliated Hospital of Nanjing University of Chinese Medicine, Nanjing 210029, China; ^2^Department of Respiratory Medicine, Jiangsu Province Hospital of Chinese Medicine, Nanjing 210029, China; ^3^Department of Physiology, Xuzhou Medical University, Xuzhou 221009, China

## Abstract

**Background:**

The efficacy and safety of combined treatment of non-small-cell lung cancer (NSCLC) using Shenyi capsules and platinum-based chemotherapy were comprehensively evaluated.

**Methods:**

A computer-based search was used to identify reports on clinical randomized controlled trials (RCTs) on this combined treatment for NSCLC from the PubMed, Embase, Cochrane Library, China National Knowledge Infrastructure (CNKI), VIP, China Biomedical (CBM), and Wanfang Data electronic databases. The databases were searched from their start to February 2020. The quality of the included studies was evaluated and then crosschecked by two independent evaluators. A meta-analysis was conducted using RevMan5.3.

**Results:**

A total of 27 RCTs involving 2,663 patients were included in the meta-analysis, including 1,380 and 1,283 patients in the treatment and control groups, respectively. The results of the meta-analysis showed that, compared to platinum-based chemotherapy alone, the 1-year survival rate (relative risk (RR) = 1.27, 95% confidence interval (CI) [1.10, 1.47], *P* < 0.01), 2-year survival rate (RR = 1.35, 95% CI [1.10, 1.65], *P* < 0.01), objective tumour remission rate (RR = 1.52, 95% CI [1.35, 1.71], *P* < 0.01), and body CD4^+^/CD8^+^ ratio (standardized mean difference (SMD) = 0.12, 95% CI [0.07, 0.17], *P* < 0.01) were increased for the combined treatment of NSCLC using Shenyi capsules and platinum-based chemotherapy; moreover, quality of life was also improved (RR = 2.09, 95%CI [1.75, 2.50], *P* < 0.01) and it reduced leukocyte toxicity (RR = 0.49, 95%CI [0.39, 0.63], *P* < 0.01), haemoglobin toxicity (RR = 0.48, 95% CI [0.28, 0.81], *P* < 0.01), platelet toxicity (RR = 0.44, 95% CI [0.28, 0.70], *P* < 0.01), vomiting reaction (RR = 0.60, 95% CI [0.45, 0.78], *P* < 0.01), and serum vascular endothelial growth factor level (SMD = −63.67, 95% CI [−67.59, −59.75], *P* < 0.01).

**Conclusions:**

The treatment of NSCLC using Shenyi capsules together with routine platinum-based chemotherapy could enhance short- and long-term efficacy, improve patient quality of life, alleviate toxicity and side-effects of platinum-based chemotherapeutic drugs, boost body immune function, and inhibit tumour neovascularisation. These findings require further validation in large-sample, high-quality RCTs.

## 1. Introduction

Lung cancer, a malignant tumour with the highest incidence worldwide, can be divided into small-cell and non-small-cell lung cancer (NSCLC) [1]. NSCLC comprises approximately 85% of lung cancer cases. The five-year survival rate of NSCLC patients is usually less than 5% because patients are usually diagnosed in advanced cancer stages and have missed their optimal chances for operation [2]. Combined chemotherapy based on platinum-based drugs is a standard treatment strategy for advanced NSCLC and is recommended by many domestic and international authorities [3]. Although chemotherapy can significantly reduce tumour volume, it is usually accompanied by severe toxicity and side-effects, with side-effects frequently seen in the haematological and digestive systems [4]. Therefore, a current research hotspot is the identification of drugs to enhance the efficacy and simultaneously reduce the toxicity and side-effects of chemotherapy [5].

In recent years, Chinese medicines have been widely used for supplementary treatment of lung cancer to significantly enhance treatment efficacy and improve patient quality of life. Shenyi capsules are the first listed inhibitors of tumour neovascularisation in China. Actually, the Shenyi capsule contains a single compound, Ginsenoside Rg3 (10 mg/capsule), a monomer of ginsenoside extracted from ginseng. Modern pharmacological studies showed that Rg3 antagonised the formation of tumour neovascularisation, inhibited tumour proliferation and metastasis, induced tumour apoptosis, and increased the radiochemotherapeutic sensitivity of tumour cells [6].

**Figure 1:**
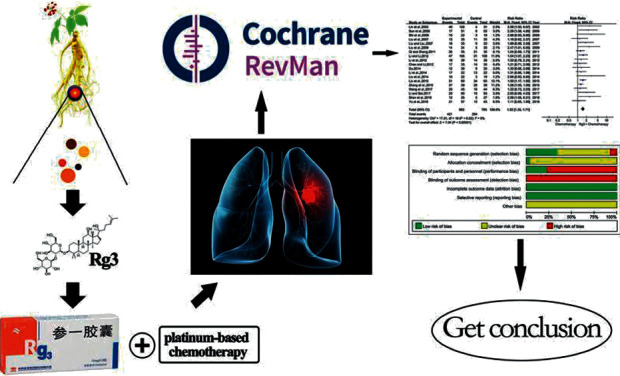
Work flow of the present study.

Increasing numbers of RCTs have confirmed the increased efficacy and decreased the toxicity of the combination treatment of NSCLC using Shenyi capsules and platinum-based drugs. However, most of these RCTs have small sample sizes and methodological problems. Therefore, the resulting conclusions have limited reference value. Although a study evaluated the efficacy and safety of the combined treatment for NSCLC in 2018, the quality of the included studies was poor and only five publications were included; furthermore, relatively fewer outcome indexes were involved [7]. Therefore, it is necessary to collect data from domestic and international RCTs on combined treatments of NSCLC using Shenyi capsules and platinum-based chemotherapy and to complete and update the clinical trials to provide current data-based evidence for the clinical application of Shenyi capsules. We report an updated and extended meta-analysis with detailed outcomes for efficacy and adverse events (Figure 1).

## 2. Materials and Methods

### 2.1. Literature Search Strategy

Studies on Shenyi capsules, NSCLC, and chemotherapy were identified by searching the China National Knowledge Infrastructure (CNKI), VIP, Wanfang Data, Chinese Biomedical (CBM), PubMed, Embase, and Cochrane Library electronic databases. Studies were searched from the start of the corresponding database to February 2020. The search terms included the English words “Shenyi capsule” and (“non-small cell lung cancer” [MeSH] or “Lung Carcinoma,” “Non-Small-Cell” [MeSH], or “Non-Small-Cell Lung Carcinoma” [MeSH] or “Carcinoma, Non-Small Cell Lung” [MeSH] or “NSCLC”) and “Chemotherapy” [MeSH] and corresponding Chinese words.

### 2.2. Inclusion Criteria

Inclusion criteria were (1) studies concerning clinical RCTs, (2) NSCLC diagnosis confirmed by cytological and pathological tests, and (3) a treatment group administered both Shenyi capsules and platinum-based drugs and a control group administered only platinum-based drugs. (4) The outcome should include at least one of the following indicators: (1) tumour objective remission rate; (2) survival rate; (3) KPS; (4) leukocyte toxicity; (5) haemoglobin toxicity; (6) platelet toxicity; (7) vomiting; (8) immune function; (9) serum VEGF. The data should have sufficient details to ensure the calculation of the risk ratios and its 95% CIs for each outcome.

### 2.3. Exclusion Criteria

The criteria for exclusion were as follows: (1) non-RCTs; (2) repeated academic articles or conference papers or duplication of periodical articles and academic dissertation; (3) inconsistent baseline study object data; (4) treatment group not administered a combined treatment of Shenyi capsules and platinum-based chemotherapy drug and lack of platinum-based chemotherapeutic drug administration in the control group.

### 2.4. Outcome Measures

Main outcome indices of efficacy were short- and long-term efficacy. The long-term efficacy was assessed by 1- and 2-year survival rates, and the short-term efficacy was assessed by using the judgement criteria for solid tumours from the World Health Organisation (WHO) [8], i.e., efficacy = complete remission (CR) + partial remission. Quality of life was determined using the Karnofsky Performance Scale (KPS) scores. An increase in KPS by more than 10 points indicated improvement, whereas a reduction by more than 10 points indicated degradation. An increase or a reduction of fewer than 10 points between pre- and post-treatment suggested stabilisation. The CD4^+^/CD8^+^ ratio in T cells of peripheral blood lymphocytes and serum vascular endothelial growth factor (VEGF) levels were assessed as immune and biochemical indicators. Adverse reactions were classified according to the toxicity classification standards of the WHO and divided into grades 0–IV. Grade II–IV toxicities were considered adverse reactions. Secondary outcome indices of efficacy were leukocyte and platelet toxicities in the digestive tract, as well as vomiting.

### 2.5. Data Extraction and Quality Assessment

The studies were independently compiled, extracted, and then crosschecked by two evaluators. Any divergence was resolved through discussion or by a third researcher. Data including the title, author, date of publication, data source, general information of the study objects, baseline data of the patient, preventative measures, and outcome index were extracted.

The methodological quality of the included studies was evaluated according to the bias risk assessment method recommended by the Cochrane Assistant Network. Seven variables including randomisation scheme, concealed grouping, double-blinding of patients and doctors, blinded assessment of the results, incomplete result data, selectively reported results, and other biases were evaluated to determine the study biases and reliability of the results.

### 2.6. Statistical Analysis

RevMan5.3 was used for data analysis. Binary variables were analysed based on the relative risk (RR) and continuous variables by mean difference. If the combined data did not have significant heterogeneity (*P* ≥ 0.10, *I*^2^ ≤ 50%), a fixed-effect model was used. If the heterogeneity was significant (*P* ≤ 0.10, *I*^2^ ≥ 50%), a random-effect model was used. Forest and funnel plots were used to assess publication bias.

## 3. Results

### 3.1. Study Identification

**Figure 2:**
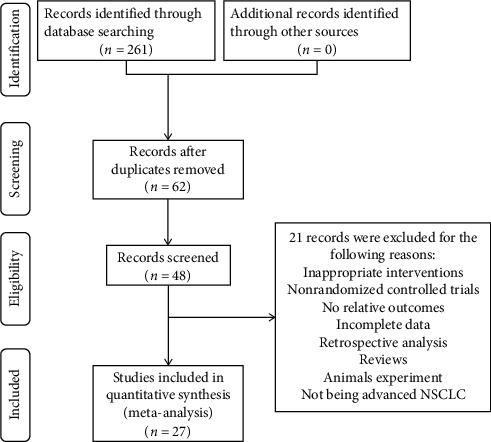
Literature retrieval and screening process.

A total of 261 studies were retrieved. After duplication removal; title, abstract, and full-text review; and consideration of the inclusion and exclusion criteria, 27 reports of RCTs involving 2,663 patients were included. A detailed flowchart that presented the process of selection is shown in Figure 2.

### 3.2. Characteristics of Included Studies

Among the studies included in the present meta-analysis, 12 administered navelbine plus cis-platinum chemotherapy, four administered taxol plus cis-platinum chemotherapy, 13 administered gemcitabine plus cis-platinum chemotherapy, three administered pemetrexed plus carboplatin chemotherapy, two administered pemetrexed plus cis-platinum chemotherapy, and one administered etoposide plus cis-platinum chemotherapy. The Shenyi capsules were administered orally. Among the 27 studies, seven did not include the KPS or PS scores, two did not indicate the neoplasm stage, and 18 studies included patients with KPS scores > 60 (Table 1).

### 3.3. Methodological Bias of the Included Studies

**Figure 3:**
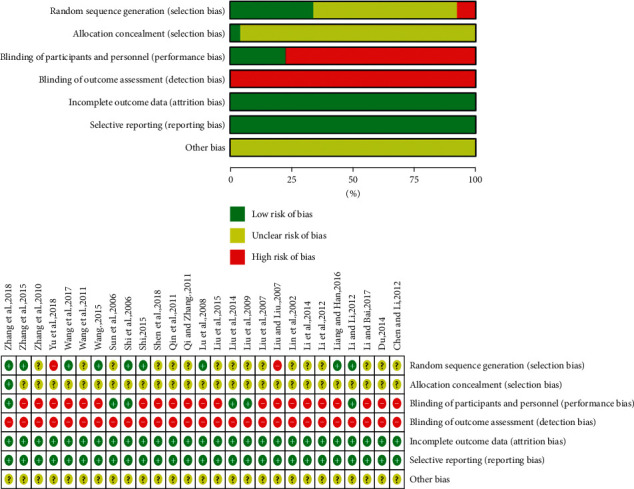
Risk of bias in included studies.

Quality assessment of the included studies. All 27 studies included in the present meta-analysis were conducted in China. Among them, nine studies used random number tables and were considered low-risk; 16 studies mentioned the word random but did not describe the randomisation method and the methods could not be confirmed by telephone or e-mail and, thus, were evaluated as unclear. Two studies used incorrect random sequence generation methods and were evaluated as high-risk. One study mentioned allocation concealment and was evaluated as low-risk. It was impossible to determine if allocation concealment was used in 26 studies; thus, they were evaluated as unclear. Six studies mentioned double-blinded methods and were evaluated as low-risk, while the 21 studies that did not mention double-blinding were evaluated as high-risk. None of the 27 studies mentioned patient drop-out and were evaluated as low-risk. The source of bias of these 27 studies could not be determined; thus, they were evaluated as unclear (Figure 3).

### 3.4. Outcome Measures

#### 3.4.1. Long-Term Efficacy

**Figure 4:**
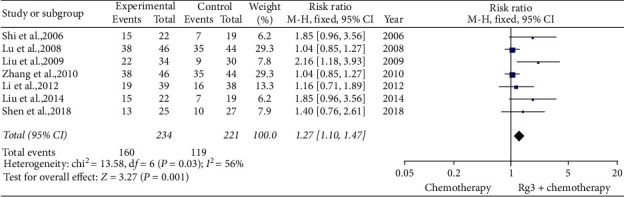
Meta-analysis of 1-year survival rate in included studies.

*(1) One-Year Survival Rate.* Seven studies [12,19,23,28–30,34] involving 455 patients compared 1-year survival rates between the treatment (*n* = 234) and control (*n* = 221) groups. The heterogeneity test showed *P*=0.03 and *I*^2^ = 56%; therefore, a fixed-effect model was used. As shown in Figure 4, the 1-year survival rate was higher in the treatment group than in the control group (RR = 1.27, 95% CI [1.10, 1.47], *P* < 0.01).

**Figure 5:**
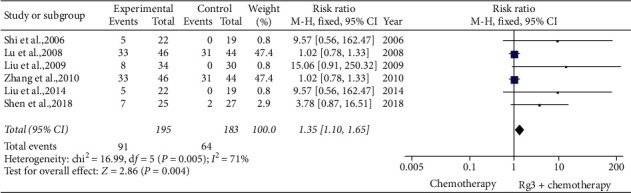
Meta-analysis of 2-year survival rate in included studies.

*(2) Two-Year Survival Rate.* Six studies [12,19,28–30,34] involving 378 patients compared the 2-year survival rates between the treatment (*n* = 195) and control (*n* = 183) groups. Heterogeneity tests showed *P*=0.005 and *I*^2^ = 71%; therefore, a fixed-effect model was used. As shown in Figure 5, the 2-year survival rate was higher in the treatment group than in the control group (RR = 1.35, 95% CI [1.10, 1.65], *P* < 0.01).

#### 3.4.2. Short-Term Efficacy

**Figure 6:**
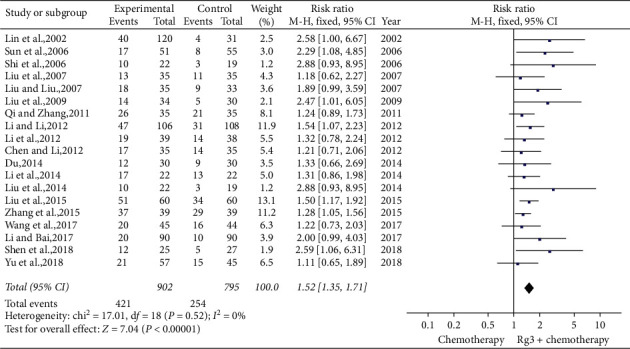
Meta-analysis of short-term efficacy in included studies.

Nineteen studies [10, 12–14, 16, 18–24, 27, 29, 31–35] involving 1,697 patients compared tumour objective remission rates between the treatment (*n* = 902) and control (*n* = 795) groups. Heterogeneity tests showed *P*=0.52 and *I*^2^ = 0; therefore, a fixed-effect model was used. As shown in Figure 6, the short-term efficacy was significantly higher in the treatment group than in the control group (RR = 1.52, 95% CI [1.35, 1.71], *P* < 0.01).

#### 3.4.3. Performance Status

**Figure 7:**
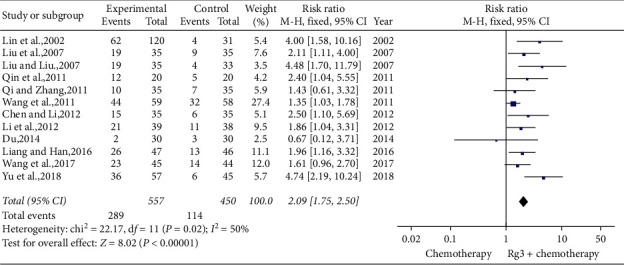
Meta-analysis of life qualities in included studies.

Twelve studies [10, 13, 15, 21, 23–27, 31, 32, 35] involving 1,007 patients compared KPS scores between treatment (*n* = 557) and control (*n* = 450) groups. The heterogeneity test showed *P*=0.02 and *I*^2^ = 50%; therefore, a fixed-effect model was used. As shown in Figure 7, the quality of life was more improved in the treatment group than in the control group (RR = 2.09, 95% CI [1.75, 2.50], *P* < 0.01).

#### 3.4.4. Toxicity and Side-Effects

**Figure 8:**
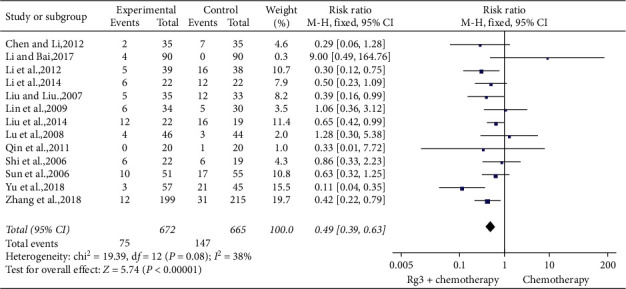
Meta-analysis of extent of leukocyte in included studies.

*(1) Leukocyte Toxicity.* Thirteen studies [9, 10, 14, 19, 20, 23, 24, 26, 29–31, 33, 34] involving 1,337 patients compared the degree of reduction in leukocyte counts between the treatment (*n* = 672) and control (*n* = 665) groups. The heterogeneity test showed *P*=0.08 and *I*^2^ = 38%; therefore, a fixed-effect model was used. As shown in Figure 8, the number of cases with more than two grades of reduction in leukocyte count was lesser in the treatment group than in the control group (RR = 0.49, 95% CI [0.39, 0.63], *P* < 0.01).

**Figure 9:**
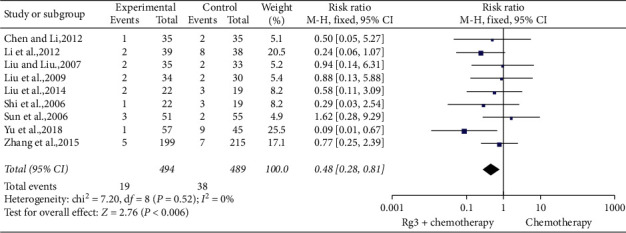
Meta-analysis of extent of haemoglobin in included studies.

*(2) Haemoglobin Toxicity.* Nine studies [9, 10, 19, 23, 24, 29, 31, 33, 34] involving 983 patients compared the degree of reduction in haemoglobin in the treatment (*n* = 494) and control (*n* = 489) groups. The heterogeneity tests showed *P*=0.52 and *I*^2^ = 0; therefore, a fixed-effect model was used. As shown in Figure 9, the number of cases with more than two grades of reduction in haemoglobin was lesser in the treatment group than in the control group (RR = 0.48, 95% CI [0.28, 0.81], *P* < 0.01).

**Figure 10:**
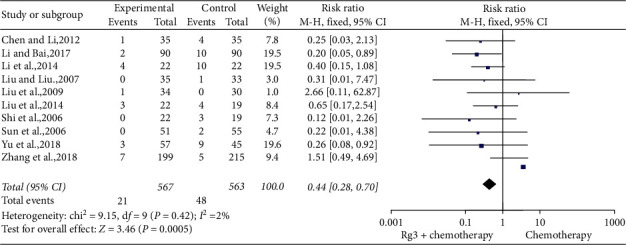
Meta-analysis of extent of platelet in included studies.

*(3) Platelet Toxicity.* Ten studies [9, 10, 14, 19, 20, 24, 29, 31, 33, 34] involving 1,130 patients compared the degree of platelet reduction between the treatment (*n* = 567) and control (*n* = 563) groups. The heterogeneity test showed *P*=0.42 and *I*^2^ = 2%; therefore, a fixed-effect model was used. As shown in Figure 10, the number of cases with more than two grades of reduction in platelet count was lesser in the treatment group than in the control group (RR = 0.44, 95% CI [0.28, 0.70], *P* < 0.01).

**Figure 11:**
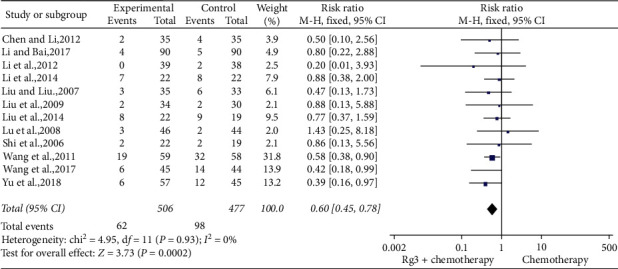
Meta-analysis of vomiting response in included studies.

*(4) Vomiting.* Twelve studies [10, 13, 14, 19, 20, 23–25, 29–31, 34] involving 983 patients compared vomiting between the treatment (*n* = 506) and control (*n* = 477) groups. The heterogeneity test showed *P*=0.93 and *I*^2^ = 0; therefore, a fixed-effect model was used. As shown in Figure 11, the number of cases with vomiting reaction above grade II was lesser in the treatment group than in the control group (RR = 0.60, 95% CI [0.45, 0.78], *P* < 0.01).

**Figure 12:**
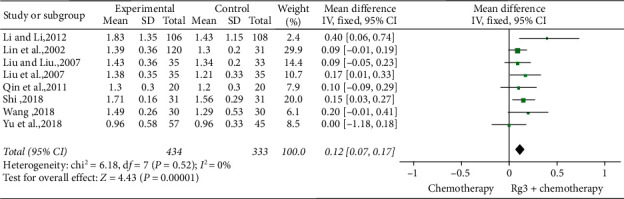
Meta-analysis of CD4^＋^/CD8^＋^ levels in included studies.

*(5) Immune Function.* Eight studies [10, 17, 22, 26, 31, 32, 35] involving 767 patients compared the CD4^+^/CD8^+^ ratio of T cells between the treatment (*n* = 434) and control (*n* = 333) groups. The heterogeneity test showed *P*=0.52 and *I*^2^ = 0; therefore, a fixed-effect model was used. As shown in Figure 12, the number of cases with reduced CD4^+^/CD8^+^ ratio was lesser in the treatment group than in the control group (standardized mean difference (SMD) = 0.12, 95% CI [0.07, 0.17], *P* < 0.01).

**Figure 13:**
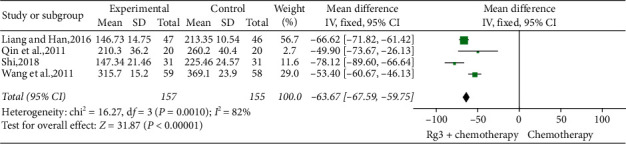
Meta-analysis of VEGF levels in included studies.

*(6) Serum VEGF Levels.* Four studies [11, 15, 25, 26] involving 312 patients compared serum VEGF levels between treatment (*n* = 157) and control (*n* = 155) groups. Heterogeneity test showed *P*=0.001 and *I*^2^ = 82%; therefore, a random-effects model was used. As shown in Figure 13, the number of cases with reduced serum VEFG level was significantly lower in the treatment group than in the control group (SMD = −63.67, 95% CI [−67.59, −59.75], *P* < 0.01).

### 3.5. Analysis of Publication Bias

**Figure 14:**
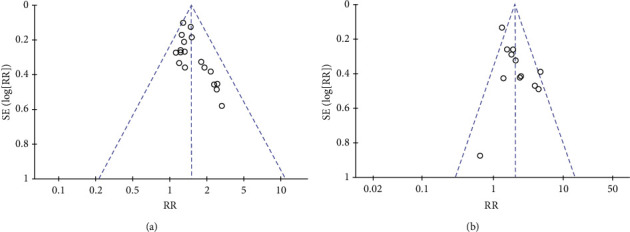
Funnel plots for publication biases in included studies: (a) objective tumour response and (b) KPS.

The short-term efficacy and quality of life reported in the included studies were subjected to funnel plot analysis (Figure 14). As shown in the scatter diagram, the top part and middle part of the 95% CI as well as the left and right sides are symmetrical, showing a normal distribution, implying a lack of publication bias.

## 4. Discussion

The incidence and death rates of lung cancer rank first among malignant tumours [36]. In 2018, GLOBOCAN estimated 2.09 million new cases (11.6% of total cancer cases) and 1.76 million deaths (18.4% of total cancer deaths). About 85% of lung cancer patients are diagnosed with NSCLC [37]. These patients are already in advanced stages of NSCLC and have missed the optimal chance for operation. At present, the combination of third-generation chemotherapy with platinum-based drug is the standard first-line strategy for NSCLC, for which a majority of patients require venous injection and hospitalisation. Although platinum-based chemotherapy can inhibit the spread of cancer cells, adverse events including toxicity and side-effects in the gastrointestinal tract and marrow are widespread [38]. These effects severely reduce immune function and degrade patient quality of life; moreover, the 1-year survival rate < 40%. Although targeted drugs can be taken orally, they are expensive and are not affordable for patients consistent with the maintenance stage.

Chinese medicines are treasures of the China civilisation and the essence of a 5,000-year culture. The ancillary efficacy of Chinese medicines for tumour treatment is definite, in addition to the limited side-effects. Although Chinese medicines are limited in their ability to shrink tumours, they help to improve patient symptoms, stabilise tumours, and improve patient quality of life, leading to long-term, high-quality survival with tumours [39,40].

Ginseng, a traditional Chinese medicine, can invigorate vital energy, restore routine pulse, rehabilitate collapse, nourish the spleen, benefit the lungs, increase intelligence, and soothe the mind. Shenyi capsule is a class I monomer anti-tumour drug in China. Its main bioactive ingredient is the monomer ginsenoside, Rg3, which is extracted from ginseng. Rg3 is a tetracyclic triterpenoid saponin. Studies have confirmed that Rg3 inhibits tumour growth. Rg3 induces apoptosis of tumour cells in their G2/M proliferative cycle and inhibits tumour neovascularisation, leading to selective inhibition of tumour cell infiltration and adhesion and inhibition of tumour metastasis. In addition, Rg3 can also regulate the immune function of the human body [41].

Several clinical studies [10,13,14,19,20,23–25,29–31,34] have shown that Shenyi capsules synergise the effect and reduce the toxicity of platinum-based chemotherapy for NSCLC. Although this treatment strategy has few side-effects and is generally well-tolerated, evidence-based data are lacking. The present meta-analysis included 27 reports of RCTs. The results showed increased clinical efficacy and quality of life in the treatment group (combined treatment using both Shenyi capsules and platinum-based chemotherapy) compared to those in the control group (treatment using only platinum-based chemotherapy). In terms of safety, the use of Shenyi capsules reduced the toxicity and side-effects of chemotherapy.

One- and two-year survival rates are both used as important indicators in RCTs. They directly reflect patient benefits of survival and are considered gold standards to assess curative efficacy. Among the studies included in the current meta-analysis, seven and six reported 1- and 2-year survival rates, respectively. Both showed positive results, suggesting that combined treatment with Shenyi capsules and platinum-based chemotherapy could increase patient survival time. This finding has significance for clinical medication.

Peripheric subgroups of lymphocytes are important indicators that can reflect general immune function, immune status, and immune equilibrium and can be used to observe efficacy and test prognosis. CD4^+^ and CD8^+^ are T lymphocytes. A reduction in the CD4^+^/CD8^+^ ratio is common in lung cancer patients after chemotherapy cycles, indicating an inhibition of the immune function in which the immune system's ability to recognise and kill mutant cells is weakening, leading to tumour growth and metastasis. Rg3, a ginseng extract and Chinese medicine, improves patient immunity. The results of the present study confirmed that Shenyi capsules could inhibit the reduction of CD4^+^/CD8^+^ ratio in NSCLC patients subjected to chemotherapy.

Angiogenesis is a marker of rapid tumour growth and metastasis, as well as an important indicator of cancer progression. VEGF can induce neovascularisation in vivo and maintain persistent tumour growth. It is the most powerful angiogenic factor found to date and an important medium for NSCLC angiogenesis. Therefore, treatment targeting antiangiogenesis agents or anti-VEGF pathways are effective approaches for the treatment of lung cancer. Research has shown that Rg3 can inhibit the cohesion, infiltration, and blood vessel penetration of tumour cells by inhibiting the generation of VEFG and further halt tumour growth and metastasis. The results of the present study confirmed that Shenyi capsules can reduce peripheral VEGF levels in patients with NSCLC.

The 27 reports of RCTs were selected according to the inclusion criteria, exclusion criteria, and quality scores. Publication bias was evaluated based on funnel plots. The indicators were evaluated using the Cochrane risk assessment table. However, the study has the following limitations:

1. The studies included in the meta-analysis were conducted in China; thus, there is some racial bias that may affect the results.
2. Most of the studies included in the meta-analysis did not describe the methods of blinding or allocation concealment, which likely led to implementation and measurement biases.
3. This study lacks reports from multicentred and large-sized RCTs. As most of the studies included in the present meta-analysis were single-centre studies with small sample numbers, there was some clinical heterogeneity that may have affected the results and the strength of the evidence.
4. No domestic or international standard of treatment is yet available for the treatment of NSCLC. Therefore, the platinum-based chemotherapeutic strategies differed between studies. Even if the same chemotherapeutic strategy was adopted, the dosage and course of treatment differed, which inevitably increases the heterogeneity of clinical studies.
5. There was publication bias among the studies included in the meta-analysis. As positive results are easier to publish than negative ones, it is challenging to find the unpublished grey literature. Moreover, less rigorous experimental design, incomplete or inconsistent preventative measures, and small sample sizes can all cause light degrees of heterogeneity. To reduce methodological heterogeneity and reporting bias and to further improve the research quality of evidence-based medicine, clinical studies should refer to high-quality experiment designs abroad, double-blinded and allocation concealment methods should be adopted, and loss-to-follow-up and drop-outs should be analysed.

## 5. Conclusions

In summary, our meta-analysis of 27 RCTs concluded that the combined treatment of NSCLC using both Shenyi capsule and platinum-based chemotherapeutical strategy is safe and effective and could enhance the short- and long-term effects of chemotherapy, improve patient quality of life, reduce the toxicity and side-effects of platinum-based chemotherapeutic drugs, increase immune function, and inhibit tumour neovascularisation. The results of this analysis were limited by the quantity and quality of the studies included in the meta-analysis; therefore, the results of the present study require validation in large-sized, multicentred, and high-quality RCTs.

## Figures and Tables

**Table 1:** Baseline characteristics of included studies.

Study	*N* (T/C)	Physical	Stage	Interventions		Outcomes
				T	C	
Zhang et al. [9]	199/215	KPS ≥ 70	III-IV	NP, TP ＋ Rg3 (40 mg/d, d1–d21)	NP, TP	(4) (5) (6)
Yu et al. [10]	57/45	KPS > 50	IIIb-IV	GP ＋ Rg3 (40 mg/d, d1–d56)	GP	(1) (3) (4) (5) (6) (7) (8)
Shi [11]	31/31	KPS > 60	Ib-IIIa	PC ＋ Rg3 (40 mg/d, d1–d28)	PC	(8) (9)
Shen et al. [12]	25/27	NR	IIIb-IV	AP, GP ＋ Rg3 (40 mg/d, d1–d56)	AP, GP	(1) (2)
Wang et al. [13]	45/44	NR	IIIb-IV	TP, PC, GP, NP ＋ Rg3 (40 mg/d, d1–d42)	TP, PC, GP, NP	(1) (3) (7)
Li and Bai [14]	90/90	NR	NR	GP ＋ Rg3 (40 mg/d, d1–d49)	GP	(1) (4) (6) (7)
Liang and Han [15]	47/46	KPS ≥ 60	IIIa-IV	GP ＋ Rg3 (40 mg/d, d1–d60)	GP	(3) (9)
Zhang et al.,[16]	39/39	KPS ≥ 70	IIIIV	NP ＋ Rg3 (40 mg/d, d1–d60)	NP	(1)
Wang [17]	30/30	NR	Ib-IIIa	AP, PC ＋ Rg3 (40–50 mg/d, d1–d60)	AP, PC	(8)
Liu et al. [18]	60/60	NR	III-IV	NP ＋ Rg3 (40 mg/d, d1–d20)	NP	(1)
Liu et al. [19]	22/19	KPS ≥ 60	III-IV	NP, TP ＋ Rg3 (40 mg/d, d1–d60)	NP, TP	(1) (2) (4) (5) (6) (7)
Li et al. [20]	22/22	NR	NR	EP ＋ Rg3 (40 mg/d, d1–d28)	EP	(1) (4) (6) (7)
Du [21]	30/30	KPS > 70	III-IV	TP ＋ Rg3 (40 mg/d, d1–d21)	TP	(1) (3)
Li and Li [22]	106/108	NR	III-IV	GP ＋ Rg3 (40 mg/d, d1–d60)	GP	(1) (8)
Li et al. [23]	39/38	KPS ≥ 60	III-IV	GP ＋ Rg3 (40 mg/d, d1–d60)	GP	(1) (2) (3) (4) (5) (7)
Chen and Li [24]	35/35	KPS > 50	III-IV	GP ＋ Rg3 (40 mg/d, d1–d21)	GP	(1) (3) (4) (5) (6) (7)
Wang et al. [31]	59/58	KPS > 60	IIIb-IV	GP, NP ＋ Rg3 (40 mg/d, d1–d56)	GP, NP	(3) (7) (9)
Qin et al. [26]	20/20	KPS ≥ 70	II-IIIa	GP ＋ Rg3 (40 mg/d, d1–d28)	GP	(3) (4) (8) (9)
Qi and Zhang [21]	35/35	KPS > 60	III-IV	GP ＋ Rg3 (40 mg/d, d1–d21)	GP	(1) (3)
Zhang et al. [28]	46/44	KPS > 70	II-IIIa	GP ＋ Rg3 (40 mg/d, d1–d56)	GP	(2)
Liu et al. [29]	34/30	KPS ≥ 60	IIIb-IV	NP ＋ Rg3 (40 mg/d, d1–d56)	NP	(1) (2) (4) (5) (6) (7)
Lu et al. [30]	46/44	KPS > 70	II-IIIa	NP, GP ＋ Rg3 (40–50 mg/d, d1–d180)	NP, GP	(2) (4) (7)
Liu and Liu. [31]	35/33	KPS ≥ 60	IIIb-IV	NP ＋ Rg3 (40 mg/d, d1–d42)	NP	(1) (3) (4) (5) (6) (7) (8)
Liu et al. [32]	35/35	KPS > 60	IIIb-IV	NP ＋ Rg3 (40 mg/d, d1–d39)	NP	(1) (3) (8)
Sun et al. [33]	51/55	KPS > 60	III-IV	NP ＋ Rg3 (40 mg/d, d1–d21)	NP	(1) (4) (5) (6)
Shi et al. [34]	22/19	KPS ≥ 60	III-IV	NP ＋ Rg3 (40 mg/d, d1–d180)	NP	(1) (2) (4) (5) (6) (7)
Lin et al. [35]	120/31	KPS > 60	II-IV	EP ＋ Rg3 (40 mg/d, d1–d42)	EP	(1) (3) (8)

*Note.* (1) T: treatment group, C: control group; Rg3 : Shenyi capsule; GP: gemcitabine + cis-platinum; TP: taxol + cis-platinum; NP: navelbine + cis-platinum; AP: pemetrexed + cis-platinum; PC: pemetrexed + carboplatin; TC: taxol + carboplatin; EP: etoposide + cis-platinum. (2) Shenyi capsule was oral administration; (3) outcome index; (1) tumour objective remission rate; (2) survival rate; (3) KPS; (4) leukocyte toxicity; (5) haemoglobin toxicity; (6) platelet toxicity; (7) vomiting; (8) immune function; (9) serum VEGF.

## Data Availability

The data used to support the finding of this study are available from the corresponding author upon request.
